# A novel single nucleotide polymorphism within the *NOD2 *gene is associated with pulmonary tuberculosis in the Chinese Han, Uygur and Kazak populations

**DOI:** 10.1186/1471-2334-12-91

**Published:** 2012-04-14

**Authors:** Mengyuan Zhao, Feng Jiang, Wanjiang Zhang, Fujian Li, Liliang Wei, Jiyan Liu, Yun Xue, Xiling Deng, Fang Wu, Le Zhang, Xing Zhang, Yuxiang Zhang, Dapeng Fan, Xiaojun Sun, Tingting Jiang, Ji-Cheng Li

**Affiliations:** 1 Institute of Cell Biology, Zhejiang University, 866 Yuhangtang Road, Hangzhou 310058, China; 2 Dongzhimen Hospital Affiliated to Beijing University of Chinese Medicine, Beijing 100700, China; 3 Department of Pathophysiology, Shihezi University School of Medicine, Xinjiang 832003, China; 4 The Fifth Hospital of Hangzhou, Hangzhou 310003, China; 5 The Sixth Hospital of Shaoxing, Shaoxing 312000, China; 6 Henan University of Science and Technology, Luoyang 471003, China

**Keywords:** *NOD2*, Arg587Arg SNP, Tuberculosis, Chinese Han, Uyghur, Kazak

## Abstract

**Background:**

The present study aimed to investigate the genetic polymorphisms in exon 4 of the *NOD2 *gene in tuberculosis patients and healthy controls, in order to clarify whether polymorphisms in the *NOD2 *gene is associated with tuberculosis.

**Methods:**

A case-control study was performed on the Chinese Han, Uygur and Kazak populations. Exon 4 of the *NOD2 *gene was sequenced in 425 TB patients and 380 healthy controls to identify SNPs.

**Results:**

The frequency of T/G genotypes for the Arg587Arg (CGT → CGG) single nucleotide polymorphism (SNP) in *NOD2 *was found to be significantly higher in the Uygur (34.9%) and Kazak (37.1%) populations than the Han population (18.6%). Also, the frequency of G/G genotypes for the Arg587Arg SNP was significantly higher in the Uyghur (8.3%) and Kazak (5.4%) populations than the Han population (0.9%). Meanwhile, no significant difference was found in the Arg587Arg polymorphism between the tuberculosis patients and healthy controls in the Uyghur and Kazak populations (*P *> 0.05) whereas, a significant difference was observed in the Arg587Arg polymorphism between the tuberculosis patients and healthy controls in the Han population (*P *< 0.01). The odd ratio of 2.16 (95% CI = 1.31-3.58; *P *< 0.01) indicated that the Arg587Arg SNP in *NOD2 *may be associated with susceptibility to tuberculosis in the Chinese Han population.

**Conclusions:**

Our study is the first to demonstrate that the Arg587Arg SNP in *NOD2 *is a new possible risk factor for tuberculosis in the Chinese Han population, but not in the Uyghur and Kazak populations. Our results may reflect racial differences in genetic susceptibility to tuberculosis.

## Background

According to the latest World Health Organization statistics, tuberculosis (TB) kills 1.7 million people each year, with 9.4 million new cases a year. It is estimated that 1/3 of the world's population has been infected with *mycobacterium *TB. In the majority of infected people, the immune response is able to adequately control the infection, and consequently only 5-10% will develop clinically active TB disease during their lifetime [1]. The incidence of TB in different ethnic groups and countries is different. In 2009, 55% of all TB cases occurred in Asia, 30% in Africa, 7% in Eastern Mediterranean Region, 4% in Europe and 3% in the Americas [2].

China has the world's second largest TB epidemic. There is a significant difference in the incidence of TB in different regions, ethnic groups and populations [3]. The TB epidemic in the Western China is higher than the national average. The prevalence rate of active, sputum smear and culture positive TB in the Uygur, Kazak and Mongolian populations of the Xinjiang Uygur Autonomous Region (Northwest China) was found to be 12.4%, 16.9% and 18.4% higher than the Chinese Han population, respectively [4]. Therefore, the differences in susceptibility to TB may be related to a genetic predisposition. Several genes such as the natural resistance-associated macrophage protein 1 [5], vitamin D receptor [6], toll-like receptor [7] and interleukin [8,9] have been associated with susceptibility to TB.

*NOD2 *(Nucleotide binding oligomerization domain containing 2) is a member of the nod-like receptor (NLR) family that acts as an intracellular pattern recognition receptor. Studies have shown that the *NOD2 *signaling pathway has a relatively independent recognition mechanism for *mycobacterium *TB [10]. *NOD2 *has been shown to recognize muramyl dipeptide (MDP), a component of bacterial cell wall peptidoglycan (PGN). Brooks et al. demonstrated that *NOD2 *controls the nature of the inflammatory response and subsequent fate of *mycobacterium *TB and *mycobacterium *bovis BCG in human macrophages [11]. Austin et al. sequenced exon 4 coding regions of the *NOD2 *gene in African Americans with TB disease and ethnically matched controlled subjects. They found that three common non-synonymous SNPs (single nucleotide polymorphisms), Pro268Ser, Arg702Trp, and Ala725Gly were significantly associated with TB disease. The Pro268Ser, Arg702Trp variations were protective against TB while, Ala725Gly polymorphism showed increased susceptibility to TB [12]. But, no association between exon 4 of the *NOD2 *gene and TB was found in the South African population [13]. Thus, polymorphisms of the *NOD2 *gene in TB may show great differences among different populations. Zhang et al. found that SNPs of *NOD2 *were associated with susceptibility to infection with *mycobacterium *Leprae [14]. In addition, 4 SNPs of the *NOD2 *have been identified to be associated with TB disease in the Chinese Han population, and one of the 4 SNPs showed a significant difference between the case and the control groups [15].

We investigated the Arg587Arg polymorphism within the exon 4 of *NOD2 *in the Chinese Han, Uygur and Kazak populations associated with TB, which was located in the winged helix and superhelical domain of *NOD2 *[16]. The difference between frequencies of *NOD2 *polymorphisms revealed susceptibility to TB in these populations and provides the experimental basis for TB risk assessment.

## Methods

### Patients and control subjects

The patients and healthy control subjects enrolled in the study were as follows: For the Han population, 219 TB patients aged 18-65 years (mean age 36.47 ± 15.73 years) from the Sixth Hospital of Shaoxing, Taizhou Shiwei Hospital and Hangzhou Red Cross Hospital, and 215 healthy controls aged 20-60 years (mean age 37.02 ± 15.29 years); for the Uygur population 86 TB patients aged 20-70 years (mean age 28.8 ± 10.1 years) from the Second People's Hospital of Aksu, Xinjiang and Wensu County People's Hospital, and 72 healthy controls aged 20-60 years (mean age 30.1 ± 8.9 years); for the Kazak population, 120 TB patients aged 20-70 years (mean age 28.8 ± 10.1 years) from the Tacheng District Hospital, Xinjiang, and 93 healthy controls, aged 20-60 years (mean age 37.02 ± 15.29 years), Table 1.

**Table 1:** Characteristics of healthy controls and TB patients in the Han, Uygur and Kazak populations

		Age (mean ± SD)	Sex (female: male)	Pulmonary TB
**Han**	TB group n = 219	18-65 (36.47 ± 15.73)	85:134	219
	Control group n = 215	20-60 (37.02 ± 15.29)	88:127	ND
	*P*	0.541^a^	0.643^b^	
**Uygur**	TB group n = 86	20-70 (28.8 ± 10.1)	37:49	86
	Control group n = 72	20-60 (30.1 ± 8.9)	31:41	ND
	*P*	0.495^a^	0.569^b^	
**Kazak**	TB group n = 120	20-70 (28.8 ± 10.1)	66:54	120
	Control group n = 93	20-60 (37.02 ± 15.29)	53:40	ND
	*P*	0.642^a^	0.536^b^	

TB = tuberculosis; ND = not determined

^a ^*P *value between total patients and controls, for *T*-test

^b ^*P *value between total patients and controls, for *χ*^2 ^test

The patients were diagnosed as having pulmonary TB based on results from acid-fast staining of sputum smear, positive tuberculin skin test and chest X-ray examination. The patients and controls were HIV negative and none was known to present any autoimmune, chronic inflammatory or any other disease conditions. All selected participants had no mixed descendants within three generations. The study was approved by the Ethics Committee of the Faculty of Medicine (Zhejiang University, China), and informed consents were obtained from all subjects before blood sampling.

### Genomic DNA extraction from blood

Genomic DNAs were extracted from the TB patients and healthy controls with the salting-out method. The frozen whole blood with anticoagulant was thawed in a 37°C water bath and 4 volumes of red blood cell lysis buffer were added for lysing red blood cells. Then, 100 μl of white lysis buffer was added for lysing white blood cells. After incubation with proteinase K at 56°C, the purified DNA was collected by ethanol precipitation. Finally, gel electrophoresis and UV spectrophotometer were used to determine the DNA concentration and purity. The sample DNA was diluted with TE buffer to make a final concentration of 100 μg/ml working solution.

### PCR amplification

Primers for exon 4 of the *NOD2 *gene were designed using the prime 5.0, which included, F1: CAGACTCAGCTTCCCAAGG; R1: AGGTGCCCAACATTCAGG (Gene ID: 64127). PCR amplification was performed using purified DNA in a 50 μl reaction at 93°C for 3 min, 95°C for 50 s, 58°C for 3 s, followed by 30 cycles at 72°C for 50 s and a final extension at 94°C for 5 min. The PCR products were sequenced using BigDye Terminator v3.1 Cycle Sequencing Kit (Applied Biosystems, Foster City, USA) and analyzed on an ABI 3730XL (Applied Biosystems, Foster City, USA) sequencer.

### Statistical analysis

The Mutation Explorer/Mutation Surveyor Version 2.2 was used for polymorphisms analysis. The comparison of age and gender between patients and controls in different populations was assessed using chi-square test and *T*-test performed with SPSS for windows (version 16.0). The genotype frequencies were assessed for Hardy-Weinberg equilibrium. The difference in allele frequencies between patients and the control groups was tested by *χ*^2^, and the genotype frequencies were analysed by Armitage Trend test and *χ*^2 ^[17]. *P *value < 0.05 was considered statistically significant.

## Results

**Figure 1:**
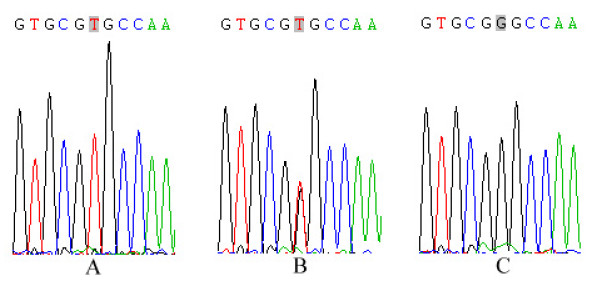
**DNA sequences of Arg587Arg in exon 4 of the *NOD2***. **A: **the genotype of T/T; **B: **the genotype of T/G; **C: **the genotype of G/G.

The exon 4 of the *NOD2 *gene was sequenced in 425 TB patients and 380 healthy controls to identify SNPs, in the Chinese Han, Uygur and Kazak populations (Figure 1). A total of 18 SNPs were found in the TB patients and the healthy controls. Except Arg587Arg SNP, the frequencies of other 17 polymorphisms were inferred lower than 1%, and we pooled all the rare variants and compared the frequencies (Table 2). The frequencies of rare variants in *NOD2 *showed no significant differences between the cases and controls (OR = 2.220, 95% CI = 0.911-5.414, *P *= 0.072). The frequencies of Arg587Arg SNPs in the Chinese Han, Uygur and Kazak patients with TB were 25%, 43% and 45.8%, respectively, whereas, the frequencies in the Chinese Han, Uygur and Kazak healthy controls were 14%, 48.6% and 39.8%, respectively (Table 3).

**Table 2:** Rare nonsynonymous and synonymous variation of NOD2 gene in the cases and control

**Amino acid variation** **(codon variation)**	**Case patients** **n = 425**	**Control subjects** **n = 380**	**OR** **(95%CI)**	***P-value***
	
Arg541Trp(CGG-TGG)	2	2		
Ala579Gly(GCC-GGC)	3	0		
Phe584Phe(TTC-TTT)	2	0		
Ala588Ala(GCG-GCA)	0	1		
Ala611Ala(GCC-GCT)	5	0		
Ala612Thr(GCG-ACG)	1	0		
Ala661Pro(GCT-CCT)	1	2		
Glu667Lys(GAG-AAG)	0	1		
Pro668Pro(CCG-CCT)	1	0		
Arg708His(CGC-CAC)	1	0		
Aal725Val(GCT-GTT)	0	1		
Pro727Pro(CCG-CCA)	1	0		
Total	17	7	2.220 (0.911-5.414)	0.072

The total occurrences of rare nonsynonymous and synonymous polymorphisms were compared between case patients and control subjects by use of a *χ*^2 ^test for 2-by-2 contingency tables.

CI, confidence interval; OR, odds ratio.

**Table 3:** The genotype frequencies of Arg587Arg (CGT → CGG) SNP of *NOD2 *gene in patients and controls of Chinese Han, Uyghur and Kazak populations

	Genotype	Patients	Controls	*Trend P-value*	OR (95%CI)
		N(Freq)	N(Freq)		
Han	T/T	162(0.74)	185(0.86)		1.00
	T/G	53(0.242)	28(0.13)	0.0023	2.16(1.31-3.58)
	G/G	4(0.018)	2(0.009)		2.28(0.41-12.63)
Uygur	T/T	49(0.57)	37(0.514)		1.00
	T/G	29(0.337)	29(0.403)	0.6575	0.76(0.39-1.47)
	G/G	8(0.093)	6(0.083)		1.01(0.32-3.15)
Kazak	T/T	65(0.542)	56(0.602)		1.00
	T/G	47(0.392)	32(0.344)	0.3838	1.27(0.71-2.25)
	G/G	8(0.067)	5(0.054)		1.38(0.43-4.46)

Genotype frequencies were compared between the patients and control subjects by Armitage Trend test and *χ*^2 ^test. N = number of alleles; Freq = frequency; CI = confidence interval; OR = odds ratio.

The G allele frequencies of Arg587Arg SNP in the Chinese Han, Uygur and Kazak patients with TB were 14%, 26%, 26%, respectively, whereas, the G allele frequencies in the Chinese Han, Uygur and Kazak healthy controls were 7%, 28%, 23%, respectively (Table 4).

**Table 4:** The allele frequency of Arg587Arg (CGT → CGG) SNP of *NOD2 *gene in the patients and controls of Chinese Han, Uygur and Kazak populations

		Allele frequency		*P-value*	OR (95%CI)
		Patients, N(Freq)	Controls, N(Freq)		
Han	T	377(0.86)	398(0.93)	0.002	2.012 (1.283-3.157)
	G	61(0.14)	32(0.07)		
HWE(P)			0.32		
Uygur	T	127(0.74)	103(0.72)	0.646	0.890 (0.498-1.401)
	G	45(0.26)	41(0.28)		
HWE(P)			1		
Kazak	T	177(0.74)	144(0.77)	0.382	1.220 (0.780-1.910)
	G	63(0.26)	42(0.23)		
HWE(P)			1		

Allele frequencies were compared between the patients and control subjects by use of a *χ*^2 ^test for 2-by-2 contingency tables. HWE = Hardy-Weinberg equilibrium; N = number of alleles; Freq = frequency; CI = confidence interval; OR = odds ratio.

The data showed that in the Han population, the G allele frequency of the Arg587Arg SNP in the patient group was twice as high than in the control group, indicating a significant difference between the two groups (*P *= 0.002). But, in the Uygur and Kazak populations, no significant difference in the G allele frequencies was observed between the patients and the controls (*P *= 0.646 and 0.382, respectively).

By comparing the TG genotype frequencies of Arg587Arg SNP in the Han population, a significant difference was observed between the patients with TB and the healthy controls (*P *= 0.0023; OR = 2.16; 95% CI = 1.31-3.58). No significant difference in the TG genotype frequencies of Arg587Arg SNP in the Uygur and Kazak populations was observed between the patients and the healthy controls (*P *= 0.6575 and 0.3838, respectively). These results indicated that the Arg587Arg SNP are associated with susceptibility to TB and may be a risk factor for the development of TB in the Chinese Han population.

## Discussion

*Mycobacterium *TB is a typical intracellular parasite. Once inside the alveoli, the MDP of the bacterial cell wall stimulate pattern recognition receptors (PRRs). The PRRs include NOD-like receptors, toll-like receptors and mannose receptors [15]. *NOD2*, a member of the NLR family, encodes a protein with caspase activation recruitment domains (CARDs) and leucine-rich repeats (LRRs). CARD interacts with receptor-interacting protein 2 (RIP2), triggering a series of downstream signal transduction pathways. This leads to the activation and translocation of nuclear factor kappa B (NFκB) into the nucleus, where it stimulates the transcription and expression of inflammatory cytokines [18-20].

It has been reported that binding of *NOD2 *and PGN breakdown products of *mycobacterium *TB can exert certain synergistic effects on tumor necrosis factor and the *NOD2 *signaling pathway has a relatively independent recognition mechanism for mycobacterium. In addition, *NOD2 *polymorphisms have been associated with susceptibility to Crohn's disease [21], inflammatory bowel disease [22], pulmonary sarcoidosis [23] and several other diseases.

Our study was the first to investigate the SNPs in exon 4 of the *NOD2 *gene among the Chinese Han, Uygur and Kazak patients with TB. We found 18 mutations and of these, the frequencies of 17 mutations were less than 1%. Only the Arg587Arg frequencies were high in the Chinese Han, Uygur and Kazak populations, and particularly higher in the Uygur and Kazak populations with TB.

Our study found that the frequencies of Arg587Arg SNP in healthy controls and TB patients were much higher in minority populations than the Han population. The frequency of G allele was also higher in minority populations than the Han population. These results indicated that there was a significant difference in Arg587Arg SNP among different ethnic groups.

Arg587Arg is located on the winged helix and super-helical domain of the *NOD2 *protein and linked to the NACHT domain [16] for synonymous mutations. In the Han population, the frequency of Arg587Arg G/T genotype was twice as high in the patient group than in the control group, indicating that the G/T genotype frequency is associated with an increased risk of TB. There was significant difference between TB patients and healthy controls. But, no significant difference of the G/T genotype frequency was observed between the TB patients and healthy controls in the Uygur and Kazak populations.

Sato et al. studied *NOD2 *polymorphisms associated with pulmonary sarcoidosis [23] and found that Arg587Arg G allele was associated with significantly better lung function parameters than the wild-type allele. In Crohn's disease, the frequency of Arg587Arg G allele was found to be much higher in the control group compared to the disease group [24]. Zhang et al. performed genome-wide association study of leprosy and identified four SNPs in *NOD2 *(rs9302752, rs7194886, rs8057341, and rs3135499) as leprosy risk factors [14]. In the present study, we found that the Arg587Arg polymorphism (CGG → TGG) have a relatively high risk for TB in the Chinese Han population (OR = 2.16, 95% CI = 1.31-3.58, *P *= 0.0023). In the African American population, three common non-synonymous SNPs in the *NOD2 *gene such as, Arg702Trp (CGG → TGG), Pro268Ser (CCC → TCC) and Ala725Gly (GCT → GGT) have been demonstrated to be associated with TB disease [12]. However, in the South African [13] and Gambian populations [25], no significant associations were observed between the *NOD2 *polymorphisms and TB disease.

Our study found that in the healthy controls, the frequency of T/G genotype in the Uygur and Kazak populations was twice as the Han population. Among Han population, the frequency of T/G genotype in the patients was twice as that of the control group. There was no significant difference in the genotype frequencies between the patients and the control groups in the Uygur and Kazak populations. However, T/G genotype was found significantly susceptible to TB in the Han population. Based on the above analysis, we believed that Arg587Arg SNP is associated with TB and is closely related to the genetic nature of the population.

The present study is the first to report that there is no significant association between Arg587Arg SNP of the *NOD2 *and TB in the Uygur and Kazak populations. But, Arg587Arg is significantly associated with the TB patients and healthy controls in the Han population and is a risk factor for TB disease in Chinese Han. The findings of this study provide data for the assessment of risk profiles regarding susceptibility to TB and also provide a molecular basis for the detection of functional changes resulting from synonymous mutations.

## Conclusions

Arg587Arg SNP in *NOD2 *is associated with susceptibility to TB and may be a risk factor for the development of TB in the Chinese Han population, but it did not show significant difference between the controls and cases neither in the Uyghur population or Kazak populations.

## Abbreviations

TB: Tuberculosis; NOD2: Nucleotide-binding oligomerization domain containing2; SNP: Single nucleotide polymorphism; NLR: Nod-like receptor; PGN: Peptidoglycan; PRRs: Pattern recognition receptors; CARDs: Recruitment domains; LRRs: Leucine-rich repeats; RIP2: Receptor-interacting protein 2; NFκB: Nuclear factor kappa B.

## Competing interests

The authors declare that they have no competing interests.

## Authors' contributions

JC Li is responsible for the experiment and paper. MY Zhao, F Jiang, JY Liu, Y Xue participated in the sample collecting and analysis of data. WJ Zhang, XL Ddeng, F Wu, L Zhang, FJ Li, LL Wei, DP Fan and XJ Sun enrolled the blood of controls and patients. X Zhang, XY Zhang and TT Jiang participated in data analysis. All authors read and approved the final manuscript.

## Pre-publication history

The pre-publication history for this paper can be accessed here:

http://www.biomedcentral.com/1471-2334/12/91/prepub
